# Targeting the sugar metabolism of tumors with a first-in-class 6-phosphofructo-2-kinase (PFKFB4) inhibitor

**DOI:** 10.18632/oncotarget.4534

**Published:** 2015-06-19

**Authors:** Jason Chesney, Jennifer Clark, Lilibeth Lanceta, John O. Trent, Sucheta Telang

**Affiliations:** ^1^ Division of Hematology/Oncology, Department of Medicine, J. Graham Brown Cancer Center, University of Louisville, Louisville, KY, USA; ^2^ Department of Biochemistry and Molecular Genetics, University of Louisville, Louisville, KY, USA; ^3^ Department of Pediatrics, University of Louisville, Louisville, KY, USA

**Keywords:** glycolysis, 6-phosphofructo-2-kinase, fructose-2, 6-bisphosphate, tumor metabolism

## Abstract

Human tumors exhibit increased glucose uptake and metabolism as a result of high demand for ATP and anabolic substrates and this metabolotype is a negative prognostic indicator for survival. Recent studies have demonstrated that cancer cells from several tissue origins and genetic backgrounds require the expression of 6-phosphofructo-2-kinase/fructose-2,6-bisphosphatase 4 (PFKFB4), a regulatory enzyme that synthesizes an allosteric activator of glycolysis, fructose-2,6-bisphosphate. We report the discovery of a first-in-class PFKFB4 inhibitor, 5-(n-(8-methoxy-4-quinolyl)amino)pentyl nitrate (5MPN), using structure-based virtual computational screening. We find that 5MPN is a selective inhibitor of PFKFB4 that suppresses the glycolysis and proliferation of multiple human cancer cell lines but not non-transformed epithelial cells *in vitro*. Importantly, 5MPN has high oral bioavailability and *per os* administration of a non-toxic dose of 5MPN suppresses the glucose metabolism and growth of tumors in mice.

## INTRODUCTION

The first committed step of glycolysis, 6-phosphofructo-1-kinase (PFK-1), functions as a metabolic sensor that dictates flux throughout the entire pathway. PFK-1 senses metabolic satiety by being tightly regulated by several products - whereas ATP, H+ ions and citrate each inhibit PFK-1, AMP stimulates PFK-1 [1-3]. In 1980, a regulatory shunt product, fructose 2,6-bisphosphate (F2,6BP), was discovered by Emile Van Schaftingen and Henri-Géry Hers to override ATP's inhibitory effects and stimulate PFK-1 in hepatocytes [4, 5]. The steady-state concentration of F2,6BP then was found to be dictated by a family of four bifunctional 6-phosphofructo-2-kinase/fructose-2,6-bisphosphatases (PFKFB1-4) that have distinct tissue distributions and kinase:bisphosphatase ratios [6]. For example, whereas the PFKFB1 family member was found to be expressed in hepatocytes and to regulate whole body glucose homeostasis, PFKFB4 was initially detected in the testes [7-9] suggesting a unique regulatory function in sperm metabolism. However, PFKFB4 was subsequently observed to be expressed in multiple organs and to be over-expressed in human tumors indicating a potential role in cancer development and/or progression [10-13].

The functional requirement of the PFKFB4 family member for neoplastic metabolism and growth then was reported in 2010 when researchers demonstrated that selective inhibition of PFKFB4 with siRNA suppressed the growth of human lung adenocarcinoma xenografts in athymic mice (U.S. patent publication #8,283,332). In 2012, two independent groups reported the results of unbiased screens for genes essential for cancer survival; they found that PFKFB4 expression was essential for the survival of glioma stem-like cells [14] and prostate cancer cells [15] but not for normal cell survival. Taken together, these recent studies suggested that PFKFB4 may be a useful molecular target for the development of anti-neoplastic agents.

PFKFB4 is a bifunctional enzyme that can increase intracellular F2,6BP and, thus, flux through PFK-1 or decrease F2,6BP and PFK-1 activity resulting in increased shunting of glucose 6-phosphate for NADPH and ribose production. Although the relative requirement of the kinase *versus* the bisphosphatase domain for cancer cell survival has been somewhat controversial [14-16], recent studies have demonstrated that: (i) recombinant human PFKFB4 kinase activity is 4.3-fold greater than its phosphatase activity; (ii) both PFKFB4-specific siRNA and genomic deletion of *Pfkfb4* result in a decrease in the steady-state concentration of intracellular F2,6BP (the product of the kinase domain); and (iii) over-expression of PFKFB4 increases F2,6BP *in vitro* [16]. Furthermore, selective inhibition of PFKFB4 expression in lung cancer xenografts causes a marked reduction in F2,6BP (rather than an increase) as well as a reduction in glucose uptake and ATP [16]. Taken together, these studies show that, in the majority of cancer cells, the kinase domain of PFKFB4 dominates to synthesize F2,6BP driving glycolytic flux into the 3-carbon portion of the pathway and enabling both ATP and anabolic substrate production. This is in sharp contrast to a potential neoplastic role for the bisphosphatase domain in suppressing F2,6BP levels and increasing flux through the oxidative pentose shunt in order to augment NADPH availability.

Based on these studies, we anticipated that pharmacological disruption of the kinase domain of PFKFB4 may decrease the glucose metabolism and growth of human cancers. We now describe the *in silico* discovery of a first-in-class PFKFB4 inhibitor, 5MPN, that reduces the steady-state concentration of F2,6BP and causes reduced glycolysis and cell cycle arrest at the G1 phase in transformed cells. 5MPN has exceptional oral bioavailability, suppresses the glucose uptake and growth of lung tumors and thus serves as an ideal lead compound for the development of test agents for phase I trials.

## RESULTS

### Discovery of a first-In-class small molecule antagonist of PFKFB4

**Figure 1:**
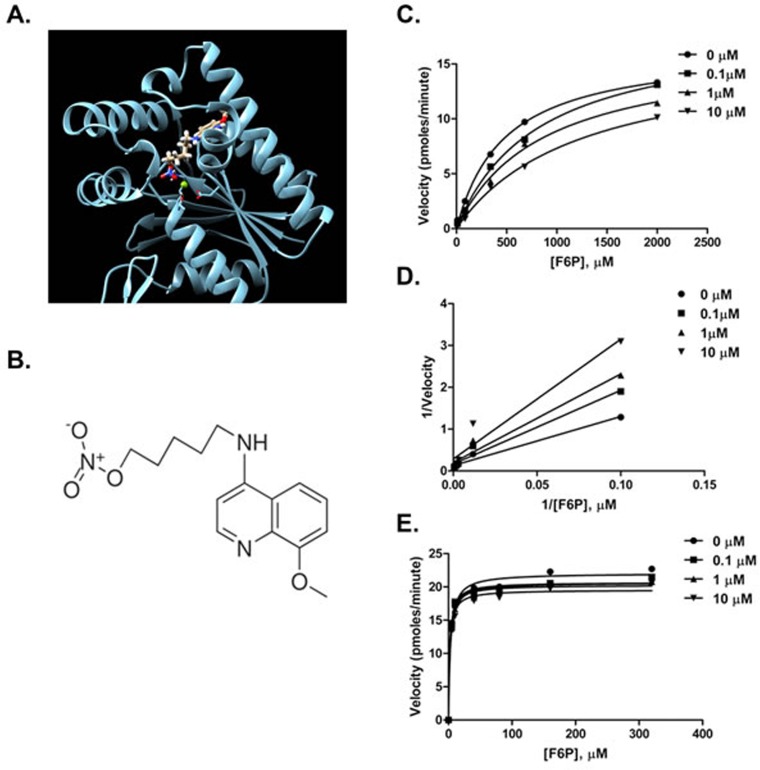
Compound 5MPN inhibits recombinant PFKFB4 enzyme activity **A**. Representation of the 5MPN molecule docked in the crystal structure of rat testes PFKFB4. 5MPN is shown in thicker stick representation than the surrounding protein residues. **B**. Molecular structure of 5MPN (MW, 305.3 Da). *In vitro* kinase assays using purified recombinant human PFKFB4 were performed as described in the presence or absence of 0.1, 1 or 10 μM 5MPN. Michaelis-Menten **C.** and Lineweaver-Burk double reciprocal **D.** plots examining PFKFB4 enzyme activity as a function of F6P concentration (0 - 2000 μmol/L) are shown. **E**. *In vitro* kinase assays using purified recombinant human PFKFB3 were performed as described in the presence or absence of 0.1, 1 or 10 μM 5MPN and the Michaelis-Menten plot examining PFKFB3 enzyme activity as a function of F6P concentration (0 - 400 μmol/L) is shown. Data shown are representative of three independent experiments.

We utilized the X-ray structure of the *Rattus norvegicus* testes PFKFB4 [17] to conduct an *in silico* screen of small molecules to identify potential compounds that may interact with the fructose 6-phosphate (F6P) binding domain of PFKFB4. Over one hundred compounds were identified, scored, ranked, and analyzed based on their association potential with the active site within PFKFB4. We physically tested the 30 best-score compounds for their ability to inhibit the kinase activity of recombinant PFKFB4. Only one of the screened compounds, 5-(n-(8-methoxy-4-quinolyl) amino)pentyl nitrate (termed 5MPN; Figure 1A and 1B), significantly inhibited PFKFB4 activity (Figure 1C). Based on Lineweaver-Burk analyses, this compound appears to be a competitive inhibitor of the F6P binding site (Figure 1D) and the Ki for 5MPN inhibition is 8.6±1.9 μmol/L. Importantly, this compound did not inhibit PFK-1 or PFKFB3 (Figure 1E) which share the identical substrate and are co-expressed with PFKFB4 in multiple cell lines and required for glucose metabolism (no inhibition of kinase activity with 10 μM). Additionally, a panel of 97 protein kinases was not inhibited by 10 μM of 5MPN providing further support for the selectivity of this compound for PFKFB4 (KINOME*scan*, *data not shown*).

### Pharmacological inhibition of PFKFB4 by 5MPN is selectively cytostatic to transformed cells

**Figure 2:**
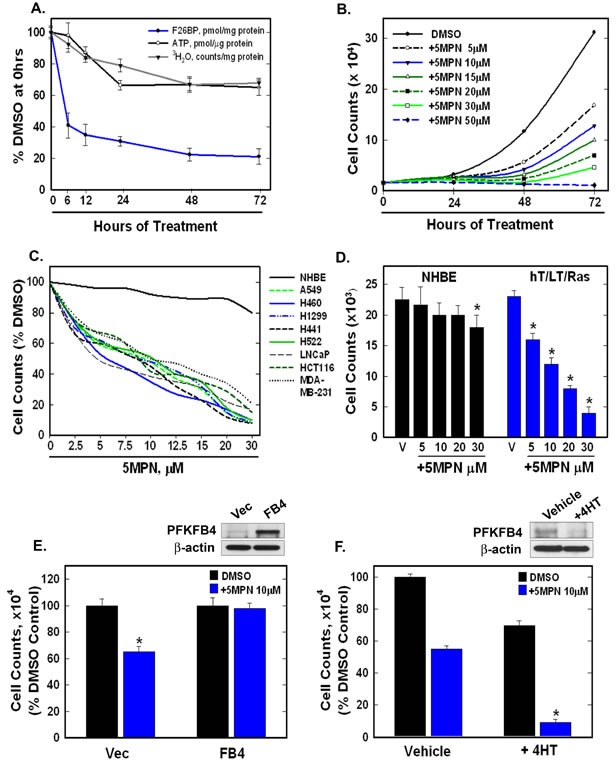
5MPN causes decreased proliferation of cancer cells preceded by a reduction in intracellular F2,6BP concentration, glycolysis and ATP **A**. H460 NSCLC cells were treated with DMSO ± 10 μM 5MPN and the effects on F2,6BP production, glycolysis and ATP were measured after 6-72 hours. **B**. Proliferation of H460 cells exposed to DMSO ± 5MPN was examined after 24-72 hours. **C**. NHBE cells and indicated transformed cell lines were exposed to DMSO ± 5MPN and viable cells counted at 48 hours. **D**. NHBE and hT/LT/Ras cells were treated with DMSO ± 5MPN and live cells counted at 48 hours (*p value <0.01 hT/LT/Ras *vs.* NHBE). **E**. H460 cells transfected with empty pCMV-XL4 (Vec) or pCMV-XL4 containing full-length PFKFB4 (FB4) for 24 hours were treated with DMSO ± 10μM 5MPN and, 24 hours later, PFKFB4 expression was examined by Western blot and viable cells were counted (*p value <0.01 Vec *vs.* FB4 exposed to 5MPN). **F**. Large T antigen-immortalized, tamoxifen (4HT)-inducible PFKFB4^−/−^ fibroblasts were exposed to vehicle (ethanol) ± 10 μM 4HT for 24 hours then treated with DMSO ± 10 μM 5MPN. Cell counts and PFKFB4 protein expression were examined 24 hours later (*p value <0.01 vehicle *vs.* +4HT, exposed to 5MPN). Data are expressed as the mean ± SD of three experiments.

H460 cells are lung adenocarcinoma cells that harbor several common oncogenic mutations (*CDKN2A*^del457^, *KRAS*^Q61H^, *PIK3CA*^E545K^, *STK11*^Q37X^) and are sensitive to inhibition of PFKFB4 using siRNA molecules [16]. We first exposed H460 cells to increasing concentrations of 5MPN and found that 5MPN led to a dose-dependent decrease in the intracellular F2,6BP concentration in these cells ([F2,6BP], pmol/mg protein at 24 hours: DMSO 6.1 ±0.2, 5μM 5MPN 2.3 ±0.05, 10μM 5MPN 1.52 ±0.2, 20 μM 5MPN 0.75±0.09, 30 μM 5MPN 0.43 ±0.1). We then examined the anti-metabolic effects of 5MPN on H460 cells over 72 hours and found that this agent first reduced the intracellular concentration of F2,6BP, glycolysis and ATP (Figure 2A) which in turn resulted in a reduction in cell proliferation (Figure 2B). We also examined the effect of 5MPN on the proliferation of non-small cell lung cancer (H460, H1299, H441, H522 and A549), breast adenocarcinoma (MDA-MB-231), prostatic adenocarcinoma (LNCaP) and colon adenocarcinoma (HCT116) cell lines and observed a dose-dependent reduction in growth over 48 hours (Figure 2C). Given that PFKFB4 has been found to be expressed by normal lung epithelia [16], we next examined the relative effects of 5MPN on normal human bronchial epithelial (NHBE) cells *versus* NHBE cells that had been sequentially immortalized with telomerase and large T antigen and transformed with H-Ras^V12^ (hT/LT/Ras cells). We found that the NHBE cells were virtually unaffected whereas hT/LT/Ras cell growth was suppressed similar to other transformed cells (Figure 2D) which we postulate may be due to the lower F2,6BP concentration in hT/LT/Ras cells relative to NHBE cells [18] in addition to an increased requirement for glycolytic flux at PFK-1. In order to interrogate the requirement of PFKFB4 inhibition for the observed suppression of proliferation (on-target effects), we next examined the effects of genetic modulation of PFKFB4 on the anti-proliferative effects of 5MPN. We found that whereas over-expression of PFKFB4 protected H460 cells from 5MPN, genomic deletion of *Pfkfb4* sensitized cells to 5MPN (Figure 2E, 2F), thus supporting the concept that inhibition of PFKFB4 by 5MPN is causing the observed reduction in H460 cell proliferation. Taken together, these data indicate that 5MPN is a potent inhibitor of PFKFB4 that selectively suppresses the proliferation of transformed cells.

### PFKFB4 inhibition with 5MPN causes a G1 cell cycle arrest that is reversed by PFKFB4 over-expression

**Figure 3:**
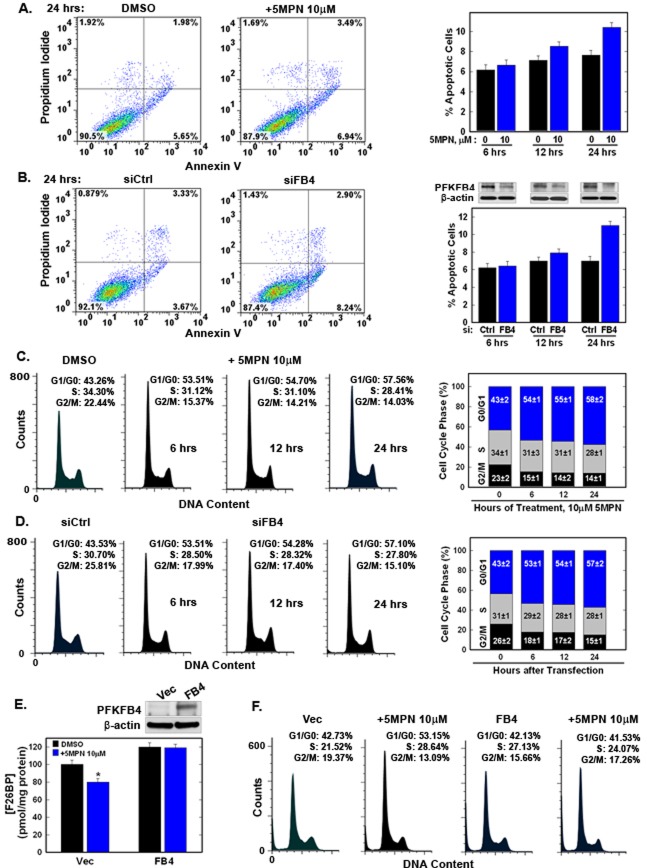
5MPN induces cell cycle arrest at the G1 phase **A, B**. H460 cells were treated with DMSO ± 10 μM 5MPN or transfected with nonsense (siCtrl) or PFKFB4 siRNA (siFB4) and were analyzed for induction of apoptosis by flow cytometry. Decrease in PFKFB4 protein expression by siFB4 was confirmed by Western blot. PI^+^ + PI/Ann V^+^ cells shown as % apoptotic cells. **C**. H460 cells were treated with DMSO ± 10μM 5MPN and distribution of cells in G1, S and G2 phases of the cell cycle was examined. **D**. H460 cells were transfected with siCtrl or siFB4 and distribution of cells in phases of the cell cycle was examined. H460 cells were transfected with empty pCMV-XL4 (Vec) or pCMV-XL4 containing PFKFB4 (FB4) for 24 hours then treated with DMSO ± 10 μM 5MPN for 24 hours and **E.** PFKFB4 protein expression and F2,6BP concentration were examined (*p value <0.01 Vec *vs.* FB4 exposed to 5MPN) and **F.** distribution of cells in G1, S and G2 phases of the cell cycle was determined. Data shown are representative of three independent experiments and are expressed as the mean ± SD of three experiments.

We noted a marked reduction in viable H460 cells after exposure to 5MPN for 48 hours (see Figure 2B) and postulated that 5MPN was inducing apoptosis, arresting cell cycle progression, or both. Whereas we observed only a minimal increase in apoptotic cells after 5MPN exposure or selective PFKFB4 siRNA transfection (Figure 3A, 3B), we observed a marked G1 arrest with both 5MPN and PFKFB4 siRNA (Figure 3C, 3D). We then over-expressed PFKFB4 and exposed the H460 cells to 5MPN at the indicated concentrations and assessed the effects on cell cycle and F2,6BP. We found that over-expression of PFKFB4 reversed the reduction in F2,6BP (Figure 3E) and G1 arrest (Figure 3F) caused by 5MPN. These studies suggest that 5MPN is suppressing PFKFB4 which in turn is resulting in a reduction in the G1/S transition.

### 5MPN has high oral bioavailability and suppresses the glucose uptake and growth of tumors in mice

**Figure 4:**
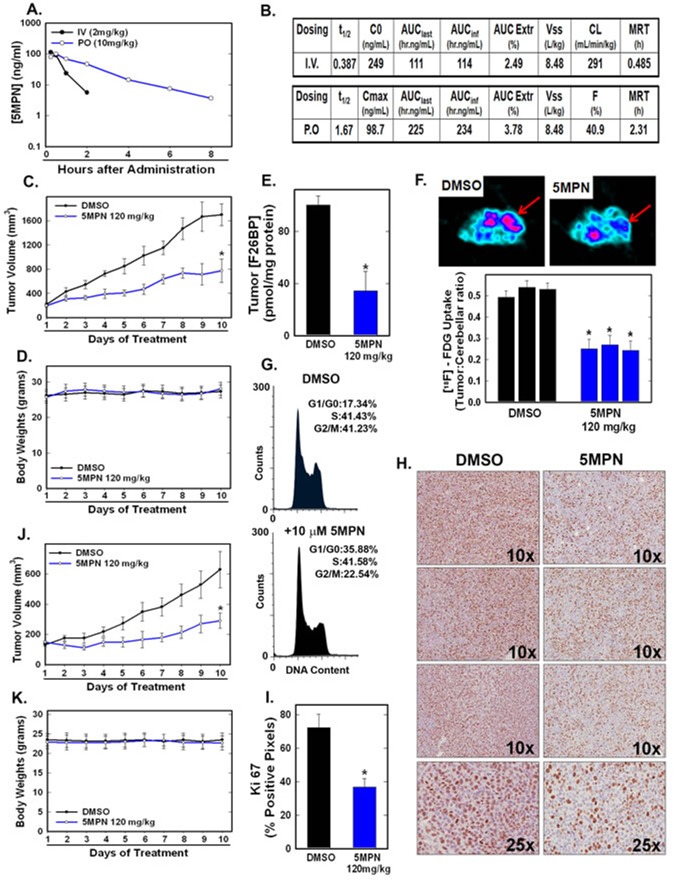
5MPN has high oral bioavailability and suppresses glucose uptake and tumor growth in mice **A, B.** Oral and IV pharmacokinetic properties of 5MPN were determined in C57BL/6 mice. Groups of 10 C57BL/6 mice were implanted with LLC cells and, when tumors reached a mass of 150-200 mg, were randomized to daily DMSO or 5MPN by gavage administration. **C, D**. Tumor and body mass measurements were collected daily. **E**. After 10 days of treatment, mice were given a dose of DMSO or 5MPN and one hour later, mice were euthanized, tumors extracted and analyzed for F2,6BP (shown as % of DMSO). **F**. Separate groups of tumor-bearing mice were administered either DMSO or 5MPN (120 mg/kg by gavage, once) and, one hour later, micro-PET scans were obtained. Regions of interest in the tumor and cerebellum were quantified in quadruplicate. Representative transverse view cuts are shown with red arrows indicating the tumor. **G**. LLC cells were exposed to DMSO ± 10 μM 5MPN and cell cycle analysis conducted. **H**. Ki67 staining of LLC tumors was examined by immunohistochemistry (representative sections shown, 10X and 25X magnification) and **(I)** Ki67-positive pixels were enumerated in a minimum of 5 fields per tumor section. Groups of 10 BALB/c athymic mice were implanted with H460 NSCLC cells and, when tumors were 150-200 mg, were randomized to daily DMSO or 5MPN by gavage. **J, K**. Tumor and body mass measurements were collected daily. *p value < 0.01 compared to controls.

We examined the pharmacokinetics of intravenous and oral administration of 5MPN and found that both routes were adequate to achieve potentially therapeutic concentrations when administered daily (Figure 4A, 4B). Given the potential usefulness of oral administration in terms of cost and convenience, we elected to pursue this route in subsequent toxicity and efficacy pre-clinical studies. Initially, we dosed C57BL/6 mice with 120 mg/kg PO (dose chosen based on a dose escalation trial) for two weeks and analyzed the effect on complete blood counts, electrolytes, hepatic and renal function, body mass and the gross and histological appearance of the brain, heart, lungs, liver, kidneys and spleen. We found no signs of toxicity either from these objective measures or from any behavioral or clinical changes (*i.e.* ruffled fur, lethargy, ataxia or labored respiration). Importantly, at this oral dose, we found that 5MPN suppressed the growth of Lewis lung carcinomas (LLC) grown in syngeneic mice (Figure 4C) and H460 human lung adenocarcinoma xenografts grown in athymic mice (Figure 4J) without affecting body weight (Figure 4D, 4K). We next examined the effects of oral administration of 5MPN on intratumoral F2,6BP and glucose uptake by LLC xenografts and observed a marked reduction in F2,6BP (Figure 4E) and 2-[^18^F]-fluoro-2-deoxyglucose uptake using positron emission tomography (Figure 4F). We confirmed that 5MPN caused a G1 arrest in LLC cells *in vitro* similar to H460 cells (Figure 4G) and then examined the number of Ki67-positive cells since Ki67 expression correlates with later S and G2 phases of the cell cycle [19]. We found that oral administration of 5MPN caused a reduction in Ki67-positive cells in the LLC xenografts (Figure 4H, 4I) suggesting that 5MPN may be reducing cell cycle progression *in vivo*.

## DISCUSSION

In this study, we report the discovery of a first-in-class and highly specific small molecule antagonist of the kinase domain of PFKFB4 that suppresses glucose metabolism and the proliferation of multiple cancer types. Importantly, 5MPN does not inhibit recombinant PFK-1 or PFKFB3 which share the substrate-binding domain and are also expressed in multiple cancer cell lines yet still reduces the glycolysis and intracellular F2,6BP of cancer cells. Furthermore, we find that the cell cycle arrest effects of 5MPN can be overcome by over-expression of PFKFB4 indicating that the anti-cancer effects of 5MPN are due, at least in part, to its inhibition of PFKFB4. Since 5MPN suppresses glycolytic flux through to the enolase reaction (Figure 2A), the availability of both fructose 6-phosphate and glyceraldehyde 3-phosphate for ribose synthesis via the non-oxidative pentose shunt is reduced by 5MPN. Accordingly, we believe that the observed G1 arrest *in vitro* caused by 5MPN (and by PFKFB4 siRNA) is a direct result of reduced availability of these glycolytic intermediates that are required for DNA synthesis during the S phase. Comprehensive metabolomic studies of the effects of PFKFB4 inhibition by genetic approaches and 5MPN will be initiated in future studies in order to better understand the observed G1 arrest caused by PFKFB4 inhibition.

We used molecular modeling to conduct virtual screens for novel ligands that might bind to the kinase as opposed to the bisphosphatase domain since the kinase activity is essential for neoplastic glucose metabolism and growth [16]. 5MPN is the first small molecule antagonist of recombinant PFKFB4 kinase activity and we found that it not only reduces F2,6BP, but also glycolytic flux through PFK-1 and cell cycle progression into the S phase. The observation that pharmacological inhibition of the kinase domain of PFKFB4 suppresses cell proliferation thus provides “proof-of-concept” that PFKFB4 kinase, as opposed to bisphosphatase inhibitors, may have utility as anti-cancer agents.

A related family member, PFKFB3, is encoded on a different chromosome [6] and has a kinase:phosphatase ratio and tissue distribution distinct from PFKFB4 [16]. A series of small molecules have been developed that selectively inhibit PFKFB3 and an optimized derivative, PFK-158, is currently undergoing phase I trial testing in advanced solid tumor patients (clinicaltrials.gov #NCT02044861). Although the relative roles of PFKFB3 and PFKFB4 are poorly understood, PFKFB4 appears to be essential for cancer cell survival and to correlate highly with hypoxic regions of tumors [16] whereas PFKFB3 localizes to both the cytoplasm and the nucleus where it activates PFK-1 and cyclin dependent kinase 1 respectively [20, 21]. We postulate that these two enzymes may provide some degree of reciprocal compensation and that dual inhibition of PFKFB3 and PFKFB4 may yield optimal suppression of intracellular F2,6BP and cell viability. Accordingly, on-going efforts are directed at understanding the relative roles of PFKFB3 and PFKFB4, and at examining the anti-tumor effects of dual *Pfkfb3* and *Pfkfb4* genomic deletion as well as combined PFK-158 and 5MPN administration.

Although there has been some degree of trepidation regarding the pharmacological targeting of enzymes that regulate an essential biochemical process such as glycolysis, we have found that PFKFB4, an enzyme expressed in several normal organs, can be pharmacologically inhibited without gross, histological or laboratory signs of toxicity. Furthermore, the observation that 5MPN was selectively cytostatic to *RAS*-transformed cells and not normal cells and suppressed tumor growth without causing toxicity is at least consistent with the hypothesis that neoplastic cells may be metabolically reprogrammed to rely more heavily on this regulator of metabolism. It is noteworthy that an inhibitor of the essential non-mutated cell cycle regulatory enzymes, CDK4 and CDK6, palbociclib, was recently found to double the progression-free survival of breast cancer patients without causing excessive toxicity [22]. Based on this recent clinical success, we predict that PFKFB4 inhibitors such as 5MPN that suppress glucose metabolism may yield favorable therapeutic indices in patients suffering with advanced solid cancers.

## MATERIALS AND METHODS

### Cell lines and cell culture

H460, H1299, H441, H522 and A549 non-small cell lung cancer (NSCLC), MDA-MB-231 (breast), LNCaP (prostatic) and HCT116 (colon) adenocarcinoma and Lewis lung carcinoma (LLC) cell lines were obtained from ATCC (authenticated by STR analysis) and were used within 6 months of acquisition. PFKFB4^−/−^ ear pinna fibroblasts isolated from TamCre/loxP/PFKFB4^−/−^ mice were immortalized as described previously [16, 23]. Normal bronchial epithelial cells (NHBE) were obtained from Lonza and NHBE cells expressing telomerase, SV40 large T antigen and activated Ras (hT/LT/Ras) were a gift from Dr. B. J. Rollins, Dana Farber Cancer Institute [24]. All cell lines were tested and found negative for mycoplasma (PCR Mycoplasma Detection Kit, ABM). Cell lines were grown in DMEM (A549, LNCaP, MDA-MB-231, LLC and PFKFB4^−/−^), RPMI 1640 (H460, H1299, H441, H522) and McCoy's 5A media (HCT116) (all from Invitrogen) containing 10% fetal calf serum (Hyclone). NHBE and hT/LT/Ras cells were grown in BEGM containing SingleQuots (Lonza). All lines were cultured at 37°C in 5% CO_2_. In certain experiments, 4-hydroxytamoxifen (4HT, Sigma-Aldrich) was added to PFKFB4^−/−^ fibroblasts at indicated concentrations.

### Cell viability

Viability was determined using trypan blue exclusion as previously described [25]. Cells were incubated in 20% trypan blue (Sigma) for 5 minutes. Cells excluding trypan blue were counted using a standard hemocytometer (Hausser Scientific) to determine total numbers of viable cells. Data are expressed as mean ± SD of three experiments.

### PFKFB4 modeling and compound screen

The PFKFB4 homology model used the rat testes PFKFB4 isozyme X-ray structure (PDB code 1BIF) as a structural template. An alignment was generated using Clustal W [26]. Four homology models were generated using Modeller [27], and the structure that best reproduced the PFKFB4 binding site was selected for further use. The residues essential to ligand binding and protein activity for PFKFB4 were correlated to equivalent residue numbers in the consensus structure. The catalytic site residues were selected to produce a residue-based protomol for Surflex 1.33 [28] for the virtual screening run using the 2007 ZINC-drug-like library containing 3,381,225 compounds. The highest-scoring 100 molecules were identified for purchase. All computational work and virtual screening was done in the James Graham Brown Cancer Center Molecular Modeling Facility. The top 30 commercially available compounds were purchased and examined for inhibitory effects on H460 cell proliferation and recombinant PFKFB4 activity.

### Transfections

For siRNA experiments, cells growing in 6-well plates were transfected with control (siCtrl, Stealth Negative Control Medium GC, Invitrogen) or PFKFB4 siRNA (siFB4, HSS107863, Invitrogen) using Lipofectamine RNAiMax (Invitrogen) and harvested as indicated. For overexpression experiments, cells were transfected with pCMV-XL4 (Vector, Vec, Origene) or pCMV-XL4 containing full-length PFKFB4 (FB4, Origene) using Lipofectamine 2000 (Invitrogen) and harvested as indicated.

### Protein extraction and western blotting

Protein extraction and blotting were conducted as previously described [16]. Membranes were probed with antibodies to PFKFB4 (Abcam) or β-actin (Sigma) followed by HRP-conjugated goat anti-rabbit or anti-mouse secondary antibodies respectively (1:5000, Pierce). Data shown are representative of three experiments.

### Kinase assays

The fructose-6-phosphate kinase activity of human recombinant PFKFB4 and human recombinant PFKFB3 in the presence of DMSO ± indicated concentrations of 5MPN was assayed as previously described [8, 16, 29]. The activity of 5MPN against 97 kinases was examined using a commercially available active-site dependent competition binding assay core service (KINOME*scan*EDGE) that quantifies the capacity of test agents to compete with an immobilized, active-site directed ligand using a DNA-tagged kinase and immobilized ligand and compound.

### F2,6BP measurements

Cells or tissues were prepared as previously described [16] and F2,6BP content measured using a coupled enzyme reaction following the method of Van Schaftingen *et al* [30] and normalized to total cellular protein measured by the bicinchoninic acid assay (Thermo Scientific). All data are expressed as the mean ± SD of three experiments. Statistical significance was assessed by the two-sample t test (independent variable).

### Glycolysis assay

Cells growing in 6-well plates were incubated in 500 μl of complete medium containing 1 μCi of 5-[^3^H]glucose per well for 60 min in 5% CO_2_ at 37°C. Media was collected, ^3^H_2_O formed via glycolysis from the 5-[^3^H]glucose measured and counts normalized as previously described [16]. All data are expressed as the mean ± SD of three experiments. Statistical significance was assessed by the two-sample t test (independent variable).

### ATP measurements

Cells were lysed and intracellular ATP determined as described previously [16]. All data are expressed as the mean ± SD of three experiments. Statistical significance was assessed by the two-sample t test (independent variable).

### Flow cytometry

To measure apoptosis, cells were stained with annexin V and propidium iodide and examined as previously described [16]. For cell cycle experiments, cells were detached, washed with cold PBS and fixed in 70% ethanol at 4°C for 30 minutes. The cells then were pelleted by centrifugation, resuspended in PBS containing PI and RNase A, incubated at 37°C in the dark for 30 minutes and analyzed by flow cytometry (BD FACSCalibur). Data were analyzed using FlowJo software (TREE STAR Inc.). Results were calculated as the mean ± SD of three experiments and data shown are representative of three independent experiments.

### *In vivo* studies

The pharmacokinetic profile was determined in female C57BL/6 mice (Charles River Laboratories) following IV and oral administration of 5MPN. Using only female mice lowered the animal numbers required for meaningful results without issues of potential gender differences in exposure. Eight time points (n=3 per time point) were used to determine indicated PK parameters calculated using WinNonLin v5.0. Plasma samples were extracted using acetonitrile and analyzed by LC/MS-MS using a PhenomexSynergi Polar-RP 4micron 50×2.0 mm column eluted with a biphasic mobile phase (0.5% formic acid in acetonitrile and water).

For xenograft studies, LLC or H460 cells collected from exponential growth phase culture were washed and resuspended in PBS. Groups of female C57BL/6 mice were injected with LLC cells (n=10 per group, s.c., 1×10^6^ cells) and groups of female BALB/c athymic mice (Charles River) were injected with H460 cells (n=10 per group, s.c, 5 × 10^6^ cells). Tumor masses were determined in a blinded fashion with Vernier calipers using the formula: mass (mg) = (width, mm)^2^ X (length, mm)/2 as previously described [31]. When tumor masses were 150-200 mg, mice were randomized to daily DMSO or 5MPN (in 30% Captisol, 100 μl, by gavage). Tumor measurements and body weights were followed daily. All data are expressed as the mean ± SD of two experiments. Statistical significance was assessed by the two-sample t test (independent variable).

At the end of the experiment, one hour after a single dose of DMSO or 5MPN, animals were euthanized, tumors removed and sections fixed in 10% formaldehyde for immunohistochemistry or snap-frozen in liquid nitrogen for analyses. Subsets of tumor-bearing mice (n=3) were administered a single dose of either DMSO or 5MPN one hour prior to imaging then injected i.p. with 2-[^18^F]-fluoro-2-deoxyglucose (FDG, 150μCi, 100 μl) and, 45 min after FDG injection, were anesthetized with 2% isoflurane in oxygen and transferred to a R-4 Rodent Scanner (CTI Concorde Microsystems) micro-positron emission tomograph to capture images as previously described [16]. Regions of interest in the tumors and cerebellum were quantified in quadruplicate and expressed as the mean ± SD of the ratio of tumor:cerebellar FDG uptake. Animal experiments were approved by the University of Louisville Institutional Animal Care and Use Committee.

### Immunohistochemistry

Five μm mounted sections of formalin-fixed, paraffin-embedded LLC tumor tissues were processed as previously described [16] and then incubated with anti-Ki-67 primary antibody (Abcam) overnight, followed by HRP-linked goat anti-rabbit secondary antibody (1:300, Pierce). Sections were developed with 3,3′-diaminobenzidine tetrahydrochloride and counterstained as previously described [16]. Slides were scanned using a ScanScope XT Digital Slide Scanner (Aperio), data analyzed with the positive pixel count algorithm (ImageScope, Aperio) and a minimum of 5 fields (20x magnification) were quantified for each tumor section. The data are depicted as % positive pixels/total pixels ± SD.
